# Supplementary material for: Patterns of Intron Gain and Loss in Fungi
Source: PLoS Biol. 2004 Nov 30;2(12):e422. doi: 10.1371/journal.pbio.0020422 (PMC532390; doi:10.1371/journal.pbio.0020422)
Supplement: Table S1 — Also available at http://genes.mit.edu/NielsenEtAl/. (4.3 MB ZIP). [file pbio.0020422.st001.zip › NielsenEtAl/html/25.html]

AN6639.1.NCU00680.1.MG02836.1.FG08851.1


```
 CLUSTAL W (1.82) Multiple Sequence Alignments - Introns Inserted


Sequence 1: MG02836.1	570 aa
Sequence 2: FG08851.1	554 aa
Sequence 3: NCU00680.1	565 aa
Sequence 4: AN6639.1	595 aa
Alignment Length: 611 aa
Number Identitical Residues: 363 aa
Alignment Score (without introns) 15909


MG02836.1 	MSAVNRTLRVAGRSLR-IQQRSS-LSTLASLNSTSALRPSSTPRGATSAFSSSVRTSSFS
NCU00680.1	MSAVNRGLRHASKQVR-LSQRVSNLSCPRFAGLQSAFRLQNSSSTSTSALP-NTRTSSFS
FG08851.1 	MSAVNRTLRTATKQLR-FQSPRG-LRVAAPLVARSALAARSSLP--------ASHGAAFS
AN6639.1  	MSASSALRRLTHRSPRNLQSRIASLSRTSCSPSSTFNSYRASSS------PALPRASRFS
          	*** .   * : :. *.:..  ..*         :      :        .:  : : **

MG02836.1 	TSTARQSSAP--NMSSAEAREYDPEISDIASYVHDTPIDSELA0FDTARWVFLDTIGCGL
NCU00680.1	TMASLQSAAPGVTPSPAAHKGYDPEITDIADYVLNKNIDSELA0FDTARWVFLDTLGCGL
FG08851.1 	TSATKFSGAP--DMSSAP-REYDPEIKDIADYVANKTIDSELA0FDTARWILLDTLGCGL
AN6639.1  	TMASLRSAAP---IPASSQVDYDPEIKDMADYIHNYKIDSDLA0FDTARLVLLDTLGCGL
          	* ::  *.**    ..:    *****.*:*.*: :  ***:** ***** ::***:****

MG02836.1 	EGLRFKECTKLLGPIVEGTTVPNGAKIPGTPYQLDPVNGAFNIGAMIRWLDYNDCWLAAE
NCU00680.1	EGLRFKECTKLLGPTVPGTVVPNGPKVPGTPYQLDPINAAFNIGTMIRWLDYNDCWLAAE
FG08851.1 	EGLRFKECSKLLGPIVPGTVVPNGPKVPGTPFQLDPVNAAFNIGAMIRWLDFNDCWLAAE
AN6639.1  	EALKFKECTKLLGPVVDGTVVPNGTRVPGTPYQLDPVNGAFNIGAMIRWLDYNDCWLAAE
          	*.*:****:***** * **.****.::****:****:*.*****:******:********

MG02836.1 	WGHPSDNLGAILAVADWVTRTNKAAAAPGSNIKPIAGGKQFTVRDVLEAMIKAHEIQGCL
NCU00680.1	WGHPSDNLGAILAVADWITRTNKAGGN-------LANGKQFTVRDVLEGMIKAHEIQGCL
FG08851.1 	WGHPSDNLGAILAVADWINRTNKAGGN-------LAGGKTFVVKDVLEAMIKAHEIQGCL
AN6639.1  	WGHPSDNLGGILAVADWVSRTNRAGGN-------IAGGKIFTIKEVLEAMIKAHEIQGVL
          	*********.*******:.***:*..        :*.** *.:::***.********* *

MG02836.1 	ALLNSYNKVGLDHVVLVKVASTAVVSKMLGLTAKQTADAVTQAWVDGQSLRTYRHTPNTM
NCU00680.1	ALLNSYNKVGLDHVVLVKVASTAVVSKMLGLNEKQTADAITQAWVDGQSLRTYRHSPNTM
FG08851.1 	ALLNSYNKVGLDHVVLVKVASTAVVSKMLGLNEKQIADAVTQAWVDGQSLRTYRHTPNTM
AN6639.1  	ALENSYNKVGLDHVVLVKVATTAVVSKMLGLSEKQTADAITQAWVDGQSLRTYRHSPNTM
          	** *****************:**********. ** ***:***************:****

MG02836.1 	SRKSWAAGDACQRAVNLALKVLKGEAGIPTVLSAPVWGFYDVLFKGKKFEFQRPYGSYVM
NCU00680.1	SRKSWAAGDACQRAVNLALKVLKGEPGVPTVLSAPVWGFYDVLFKGKKFEFQRPYGSYVM
FG08851.1 	SRKSWAAGDACQRAVNLALKVLKGEQGVPTVLSAPVWGFYDVLFKGQKFEFQRPYGSYVM
AN6639.1  	SRKSWAAGDACQRAVNLVLKVQKGEGGLKTVLSAPVWGFYDVLFKGNKFKFQRPYGSYVM
          	*****************.*** *** *: *****************:**:**********

MG02836.1 	ENVLFKVSYPA1EFHSQTAVEASQKIHDQLKAMGKSAADIKEITCRTHEACVRIIDKQFK
NCU00680.1	ENVLFKVSYPA~EFHSQTAVEASEKIHAQLKAMGKSAADIKEVTIRTHEACVRIIDKQFK
FG08851.1 	ENVLFKVSYPA1EFHSQTAVEASEKIHHLLKSQGKSAADIKSITCRTHEACIRIIDKQFK
AN6639.1  	ENVLFKVSYPA1EFHSQTAIEAAEIINKKLAALGKSAKDIKEVTNRTHEACIRIIDKQFK
          	*********** *******:**:: *:  * : **** ***.:* ******:********

MG02836.1 	PMDNFADRDHCIQ~YMCSVMLVFGRLTAGDYVDGSEAATSPLVESLRKKIRCVEDKQFTA
NCU00680.1	PMDNFADRDHCIQ~YMCSVMLVFGRLTAGDYTDGSEAATSELVESLRKKIKCVEDPQFTA
FG08851.1 	PMDNFADRDHCIQ~YMCATMLVFGRLEATDYTDGGEAATSPLVESLRQKIKCVEDPQYTK
AN6639.1  	AMDNFADRDHCVQ0YMVATMLVFNRLTANDYADGSEAATSPLLEDLRKRIRCVEDPKFTL
          	.**********:* ** :.****.** * **.**.***** *:*.**::*:**** ::* 

MG02836.1 	DYHDPKLRTISNGLTVELNDGTFLDEVVIEAPLGHRLRREEAKPEILSKFKRHLEAHYPA
NCU00680.1	DYHDPALRTISNGLTVELNDGTFLDEVVVEAPLGHRLRREEAKPHILAKYKRHLEPHFSA
FG08851.1 	DYHDPKLRTISNALTVELNDGTVLDEVAVEAPLGHRLRREEAKPVILEKYKRHLGPHYPE
AN6639.1  	DYHDPSKRTIPNALTVTLNDGTVLDEVVVEAPLGHRLRRDEAKPEILDKYKRHLQAHFDQ
          	*****  ***.*.*** *****.****.:**********:**** ** *:**** .*:  

MG02836.1 	AKVQELIDLGMDAKKLEATSVDEYVDMYVVK-------~-------DSKFVQ----~---
NCU00680.1	DKVEKLVALGQNPKELEAMSVDEYVDLYVNE-------~-------KSEFAQ----~---
FG08851.1 	ARVKELVELNLDAKKLESTPVDEYVDLYTVE-------~-------SSKFVQ----~---
AN6639.1  	ARLQELVDLGNNKAELEAYEVDKYVDLYVRDKIVAPTA2QTDKSYTDTKGLQTEVI0PNG
          	 ::::*: *. :  :**:  **:***:*. ..  :.:: .:..: :.::  *:.   ...

MG02836.1 	----------------
NCU00680.1	----------------
FG08851.1 	----------------
AN6639.1  	GVEPPTLRLRVSRSAD
          	. ...:     : ::.
```
